# Unveiling the Burden: A Six-Year Retrospective Analysis of Pressure Ulcer Epidemiology in a ICU

**DOI:** 10.3390/nursrep14040239

**Published:** 2024-11-01

**Authors:** Sofia Vieira, António Mostardinha, Paulo Alves

**Affiliations:** 1 Center for Interdisciplinary Research in Health|CIIS, Wounds Research Lab, Faculdade de Ciências da Saúde e Enfermagem, Universidade Católica Portuguesa, 4169-005 Porto, Portugal; amostardinha@ucp.pt (A.M.); pjalves@ucp.pt (P.A.); 2 Intensive Care Department, Hospital Dr. Nélio Mendonça, Serviço de Saúde da Região Autónoma da Madeira, EPERAM, 9000-177 Funchal, Portugal; 3 Physical Medicine and Rehabilitation Department, Unidade Local de Saúde do Alto Ave, 4835-044 Guimarães, Portugal

**Keywords:** critical care, critically ill patients, epidemiology, nursing, pressure ulcers

## Abstract

Objective: This study describes the epidemiological changes in pressure ulcers (PUs) in a Portuguese intensive care unit (ICU) from January 2017 to June 2023, characterizes critically ill patients with PUs, identifies specific risk factors, and assesses the effectiveness of implemented preventive measures. Materials and Methods: A retrospective observational cohort study was conducted, analyzing records of ICU patients with PUs during the specified period. Data were extracted from the institution’s Global Risk Management application and the ICU’s electronic PU registry. The study included patients with ICU stays longer than 24 h and excluded those with ineligible clinical records or incomplete characterization data. Results: Among 3816 evaluated patients, 257 developed a total of 345 PUs, averaging 1.4 PUs per patient. The average PU prevalence rate was 6.81%, with the highest prevalence in 2020 (11.0%) and the lowest in 2022 (3.48%). The average incidence rate was 3.76%, peaking at 5.71% in 2020 and declining to 2.54% in 2023. The sacrum and heels were the most commonly affected areas, with Category 2 PUs being the most frequent. Key intrinsic risk factors included systemic diseases and sensory deficits, with pressure identified as a significant extrinsic factor. Preventive measures focused on risk assessment and pressure control. Conclusions: The study reveals a PU prevalence of 6.81% and an average incidence of 3.76%, underscoring the need for enhanced preventive strategies, especially in anatomical areas like the sacrum and heels. It emphasizes the importance of personalized assessments, continuous education for nursing staff, and a multidisciplinary approach to improve patient outcomes and care quality in the ICU.

## 1. Introduction

Pressure ulcers (PUs) are key quality indicators in healthcare and a major concern in intensive care units (ICUs) due to their significant impact on both patient outcomes and healthcare systems [1,2]. They are among the most frequent, costly, and yet potentially preventable adverse events. For ICU patients, PUs can lead to reduced quality of life and increased mortality risk, highlighting the importance of their prevention and management [1,2]. Identifying the risk factors associated with their development is crucial to mitigate this burden and improve patient outcomes. However, current literature on PU management reveals significant gaps, particularly regarding the effectiveness of nurse training programs and preventive interventions in critically ill patients. Many studies focus on intervention bundles and protocols but show inconsistencies in their application and lack comprehensive evaluations of their impact, especially in ICUs [3,4,5]. Additionally, there is no unified approach that integrates the multiple factors influencing PU development in ICUs into a tailored preventive strategy.

PUs develops through a complex process of damage to the skin and/or underlying tissues due to pressure or a combination of pressure and shear forces, typically over bony prominences or associated with medical devices [6,7,8]. The prevalence of hospital-acquired PUs is particularly concerning in ICU settings, where critically ill patients are highly vulnerable due to factors such as the complexity of their health conditions, limited mobility, invasive mechanical ventilation, vasopressor use, and the presence of invasive medical devices [3,6,9].

Pre-COVID-19 epidemiological studies indicated that the prevalence of PUs in adult ICU patients ranged from 16.9% to 23.8%, with cumulative incidence varying between 10% and 25.9% [10,11]. Some studies even reported an incidence as high as 41.4% [10]. These statistics highlight the urgent need for targeted, proactive, and multifaceted preventive approaches for ICU patients, whose complex medical conditions and immobility heighten their susceptibility to PUs [12]. Implementing comprehensive care approaches is crucial for effectively mitigating the significant risks and burden associated with PU development in this vulnerable population. 

The COVID-19 pandemic significantly transformed the epidemiology of PUs. Healthcare facilities faced complex challenges in managing PUs due to the influx of COVID-19 patients and the rapid adaptation of structural, environmental, material, and human resources to accommodate the surge in hospitalizations. Given the historical data on PU development in critical care settings, a significant increase in incidence during the pandemic was anticipated.

The unique clinical features of COVID-19, including extend hospitalizations, reduced mobility due to severe illness, increased reliance on mechanical ventilation, and the necessity for prone positioning to support respiratory function, emerged as new contributing factors to PU development [13,14,15]. The complex interplay of these factors, including direct tissue deformation, inflammatory damage, and ischemic insults, exacerbated the risk of PU formation in this vulnerable patient population. 

New factors emerged as contributors to PU development, including prolonged hospital stays, limited mobility due to severe illness, mechanical ventilation, and the need for prone positioning for respiratory support. These elements were associated with three key etiological factors: direct deformation damage, inflammatory damage, and ischemic damage, all of which contribute to the degradation and deterioration of cells and tissues [16]. The concept of individual susceptibility to PUs emerged, which depends on the integrated functions of the body system, which is dynamic and extremely challenging to predict in critically ill patients [16].

Additionally, the implementation of infection control measures, such as the mandatory use of personal protective equipment for healthcare workers, coupled with reduced nurse-patient ratios due to staffing shortages, and the redirection of health resources and budgets away from routine preventive care to manage the influx of COVID-19 patients, has significantly hindered the effective preventive care and management of PUs during this period. The increased workload, limited resources, and shifting priorities have resulted in decreased attention and resources allocated to PU prevention strategies, thereby exacerbating the burden on both patients and the healthcare system [15]. 

The pandemic profoundly transformed key aspects of PU prevention, influenced by the pathophysiology of the COVID-19 virus, the intrinsic conditions of the patients (such as systemic coagulopathy and hypercoagulable states affecting the skin), and the extrinsic factors of the care environment (prone to overloading healthcare systems). Consequently, the preventive measures that were effective prior to COVID-19 may not have been practical or feasible during the pandemic [15].

As healthcare systems begin to recover from the pandemic, a new dynamic in PU epidemiology is anticipated. This shift underscores the importance of healthcare institutions reassessing and adapting their practices to effectively respond to emerging challenges [15]. Current knowledge of PU aetiology reinforces that the development of these lesions is primarily due to sustained mechanical load, which leads to the deformation of soft tissues. Tissue deformation can be minimized by reducing peak deformation and stress on tissues using specialized support surfaces and effective pressure redistribution techniques [16,17,18,19]. 

Early intervention is critical, as it can prevent the progression of microscopic tissue damage into clinically significant pressure ulcers. This proactive approach is vital for reducing incidence rates and enhancing patient outcomes. By implementing strategies that focus on both minimizing tissue deformation and increasing tissue tolerance, healthcare providers can work to mitigate the burden of PUs, particularly in critical care settings where patients are at heightened risk due to their complex medical conditions and limited mobility [16,17,18,19].

Furthermore, adopting additional preventive strategies aiming to increase tissue tolerance should be adopted, such as optimizing patients’ nutrition and providing comprehensive skin care [18]. These innovations, including the use of special mattresses and regular skin inspections, are both promising and urgently needed, especially given that PU incidence and prevalence remain high across all healthcare settings, particularly in ICUs where patients are at an elevated risk due to their critical condition and limited mobility [4,20].

In this context, the primary objective of this study is to provide a comprehensive epidemiological assessment of PUs among critically ill patients treated in the ICU over the six-year period, from January 2017 to June 2023. The research aims to thoroughly characterize the demographic and clinical profiles of these patients, with an emphasis on identifying risk factors for PU development, by analyzing intrinsic and extrinsic factors, and evaluating the adequacy of preventive measures. 

Furthermore, the study will critically assess the effectiveness of current preventive measures, particularly the use of specialized support surfaces, to identify successful approaches and areas for improvement. 

Utilizing a retrospective design, the research will analyze existing patient data to evaluate trends in PU incidence and contributing factors, providing insights into the challenges of PU prevention and management in ICU settings, especially during the COVID-19 pandemic.

The findings will serve as a valuable resource for healthcare providers, policymakers, and researchers, aiming to enhance knowledge of PU management in critical care and improve patient safety and outcomes. 

## 2. Materials and Methods

### 2.1. Design, Context and Sample

This retrospective observational cohort study is based on health records from the ICU of a central hospital within the Serviço de Saúde da Região Autónoma da Madeira, EPERAM (SESARAM, EPERAM). The ICU admits approximately 650 admissions annually, delivering high-complexity intensive care to critically ill patients in need of rigorous monitoring and specialized care. 

The study population was selected through a non-probability convenience sampling approach, including all adult patients who presented with PUs either upon admission or acquired during their hospitalization between 1 January 2017 and 30 June 2023. Patients with ICU stays longer than 24 h were included in the study, while those with ineligible clinical records or incomplete characterization data were excluded from the analysis.

The study was authorized by the Ethics Committee for Health and the Scientific and Research Committee of SESARAM, EPERAM, ensuring proper ethical oversight and approval for the retrospective data collection. Given the retrospective nature of the data collection, the ethics committee determined that informed consent from participants was not required.

The ICU does not have a dedicated PU prevention protocol but adheres to institution-wide guidelines based on directives from the Directorate-General of Health (Direção-Geral da Saúde) [21]. The PU prevention measures implemented in the ICU include comprehensive risk assessment within the first 12 h of admission, with daily reassessment thereafter. Skin assessment and care are conducted upon admission and at least once per nursing shift. Pressure-reducing measures are tailored according to each patient’s risk profile. To further enhance prevention efforts, all ICU areas have been equipped with specialized support surfaces, including optimized intensive care beds and immersion system mattresses, designed to minimize the risk of pressure-related tissue damage. This investment reflects an ongoing commitment to enhancing care practices and improving patient outcomes.

### 2.2. Data Collection and Analysis

Data for the study were collected retrospectively, spanning the period from 1 January 2017 to 30 June 2023. The six-year period was a logical and valuable choice for the research because it allowed for the observation of trends and patterns in the incidence and prevalence of PUs, including the impact of significant events such as the COVID-19 pandemic. Additionally, the aim was to have a larger sample, ensuring statistically significant results and enabling the evaluation of the effectiveness of interventions and changes in care practices. 

For the initial years (1 January 2017, to 31 December 2019) data were obtained from the ICU’s dedicated wound care liaison nurse, who diligently maintained the unit’s electronic PUs registry. This registry contained comprehensive records of pressure ulcer occurrences, including detailed documentation of the development, characteristics, and treatment of these wounds. From 2020 onwards, data were provided by the hospital’s statistics department and the global risk management committee, utilizing the robust RISI platform for managing risk and quality across the healthcare organization. These complementary data sources ensured access to thorough and reliable information, including detailed nursing records of PU notifications and overall ICU admission caseloads during the study period.

Data were pseudo-anonymized to protect patient privacy, and the process involved replacing identifiable information with unique codes that maintained confidentiality while allowing for data analysis. The pseudo-anonymized data were securely transferred into a Microsoft Excel spreadsheet and subsequently organized into IBM SPSS version 28.0 data files for comprehensive statistical analysis. The research team carefully reviewed the data, ensuring that any information not explicitly documented in the records was excluded from the study, and no assumptions were made regarding undocumented details.

The collected data included detailed demographic information, such as age, gender, and comorbidities; comprehensive clinical data, including diagnoses, treatments, and medical history; epidemiological data, such as incidence and prevalence of PUs; and documented risk assessments, such as the Braden scale score at the time of PU notification. The Braden Scale [21] was used to assess the risk of developing PUs based on intrinsic factors (e.g., impaired mobility, nutritional status, and skin integrity) and extrinsic factors (e.g., pressure, friction and shear).

### 2.3. Statistical Analysis

Descriptive statistical analysis involved calculating various measures to summarize the data, including means and standard deviations for quantitative variables to describe central tendency and dispersion. Frequency analysis was conducted for categorical variables to determine their distribution and calculate the corresponding proportions, providing insights into the characteristics and patterns present in the collected data.

Qualitative data were described by presenting the counts and percentages of the different categories, while quantitative variables were summarized using measures of central tendency (mean) and dispersion (standard deviation). To examine the relationships between qualitative variables, the Chi-square test was employed, a statistical technique widely used for analyzing the associations between categorical variables.

The PU incidence rate was calculated as the ratio of the number of patients who developed PUs during a given period to the total number of patients at risk of developing PUs during the same period. A higher incidence rate indicates a greater risk of PU development in the studied population. Additionally, the PU prevalence was determined as the proportion of patients with existing PUs at a specific point in time, regardless of when the ulcers developed. 

The PU prevalence rate was calculated as the ratio between the number of patients with existing PUs during a given period and the total number of patients at risk. A higher prevalence rate indicates a greater overall burden of PUs within the patient population.

For the years with missing data (e.g., 2023), appropriate statistical methods were employed to address this gap, ensuring that the analysis remained robust and reflective of the overall trends. The severity of PU was classified according to established clinical criteria, categorizing them from Stage I (non-blanchable erythema of intact skin) to Stage IV (full-thickness skin and tissue loss) [6].

All statistical analyses for this study were conducted using IBM SPSS version 28.0 software, with a significance level for all statistical tests set at 0.05, a commonly used threshold to determine the probability of observed results occurring by chance.

## 3. Results

### 3.1. Demographics

Out of the 3816 patients admitted to the ICU between January 2017 and June 2023, a total of 257 adult patients met the study’s eligibility criteria. These patients had a combined total of 345 recorded PUs. The cohort consisted predominantly of males, accounting for 67.7% (*n* = 174) of the eligible patients, with an average age of 65.7 years. Most patients (94.2%) were assessed as being at high risk for PU development upon registration, indicating a vulnerable population.

The data in Table 1 offers valuable insights into the demographics and distribution of PUs among ICU patients over the analyzed period. Notably, 52.5% of all PUs originated within the ICU. However, analyzing the annual distribution, we verify that in 2019 and 2020, a significant proportion of PUs developed in other hospital departments, accounting for 48.6% and 50.0%, respectively. This shift highlights the importance of extending preventive measures beyond the ICU to encompass the entire hospital environment. It is also worth noting that 2% (*n* = 7) of patients lacked recorded data regarding the origin of their PUs.

In terms of patient demographics, most PU cases occurred among male patients, with a steady increase in high-risk assessments over the years, reaching 100% in 2020. The highest percentage of male patients affected was recorded in 2019 (82.7%), while the lowest was observed in 2022 (45.5%). High-risk patients, identified by a Braden score below 16, are more susceptible to skin breakdown due to factors such as reduced mobility and increased moisture exposure. In contrast, patients with a Braden score above 16 are considered at lower risk, although continuous monitoring remains essential to prevent PU development.

The data also reflects the evolving nature of PU management, as demonstrated by variations in age and the number of PUs per patient. While most patients typically developed only one PU in the ICU, with a peak of 92.9% in 2020, there was a sharp increase in 2023, where 44.4% of patients developed multiple PUs, signaling the growing complexity of care in this patient population.

These findings emphasize the increasing challenges of PU prevention and management, particularly in a high-risk and aging ICU population. The rise in high-risk assessments, coupled with the significant shift in the location of PU development in 2019 and 2020, calls for comprehensive, hospital-wide preventive strategies. Addressing these trends will require dynamic interventions that are not only focused on the ICU but also ensure continuity of care across all hospital departments.

### 3.2. Prevalence Rate, Incidence and Characteristics of PU Acquired in ICU

Between 1 January 2017 and 30 June 2023, a total of 345 PUs were recorded in the ICU of HDNM, with 181 of these PUs originating within the ICU itself. The prevalence and incidence rates of PUs fluctuated significantly over the study period, revealing crucial insights into the ICU’s PU management.

The data from Table 2 highlights important trends in the prevalence and incidence of PUs in the ICU over the study period, with notable fluctuations that merit further exploration. The average prevalence rate was 6.81%, with a minimum of 3.48% in 2022, and a maximum of 11.0% in 2020. Similarly, the incidence of PUs showed an average of 3.76%, with a range from 2.54% in 2023 to a peak of 5.71% in 2020.

**Figure 1:**
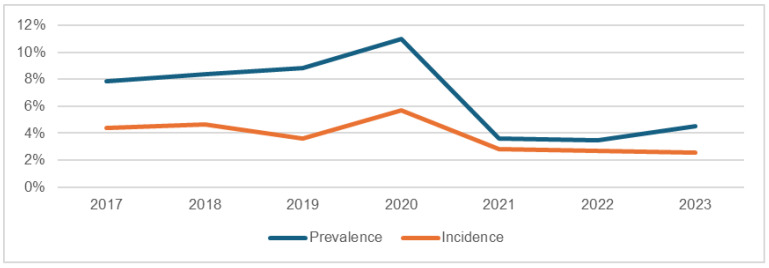
Distribution chart of incidence and prevalence in the ICU.

This spike in 2020 (Figure 1) suggests a significant disruption in PU prevention efforts, likely exacerbated by the global COVID-19 pandemic. The increased use of prone positioning for respiratory management, extended ICU stays, and the overwhelming strain on healthcare resources during the pandemic could have contributed to this rise in prevalence and incidence. 

The decline in incidence after 2020 suggests that preventive measures were gradually restored and optimized as the immediate pressures of the pandemic eased. However, the persistence of relatively high prevalence rates, even in subsequent years, indicates that the residual effects of the pandemic, along with an aging and high-risk ICU population, continue to present challenges for PU prevention and management.

**Figure 2:**
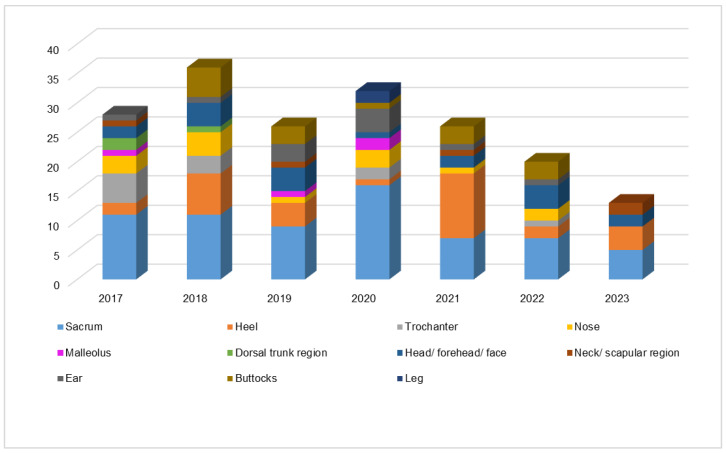
Graph of UP distribution acquired in the ICU according to Anatomical Location.

Most PUs were localized to the sacral region and heels, areas particularly vulnerable in bedridden patients (see Figure 2). The sacral area is a critical pressure point when patients lie supine, while the heel’s bony prominence makes it highly susceptible to ulcers, especially in individuals with limited mobility or comorbidities like diabetes.

Over the years, an increase in PUs in the head, forehead, and face regions has been noted, likely due to prolonged unconsciousness and the use of medical devices, such as oxygen masks and ventilation equipment, which place pressure on these areas. Patients with respiratory comorbidities are especially vulnerable, as they frequently require extended periods of mechanical ventilation. Understanding the relationship between PU locations and patient comorbidities is crucial for effective prevention. 

**Figure 3:**
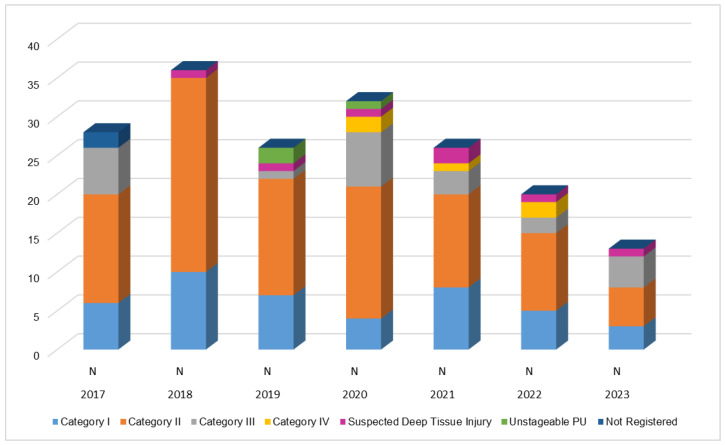
Chart of PU distribution acquired in the UCI according to Category.

Throughout the study, Category 2 PUs were the most frequent type, characterized by partial-thickness skin loss with exposed dermis (see Figure 3). 

The predominance of PUs with an area of ≤5 cm^2^ suggests that many cases are identified at an early stage of ulcer development. However, limitations in the available data may restrict our ability to fully understand the extent and implications of these findings.

### 3.3. Analysis of the Occurrence of PU in the ICU

To characterize critically ill patients with PUs, particularly in exploring the relationship between gender, age, and PU risk over the analyzed years, the Chi-square test of independence was employed. As indicated in Table 3, while no significant associations were found among the studied variables, notable trends emerged. For instance, in 2018, a higher, though not statistically significant, incidence of PUs was observed in females (21.7%). Similarly, during 2019 and 2020, older patients demonstrated an increased, yet non-significant, incidence of PUs, particularly those aged ≥72 years, with rates of 18.8% and 22.9%, respectively. This trend is concerning as it aligns with the observed peak in PU occurrence during these years, highlighting a potential vulnerability among the older demographic.

Furthermore, during this same period, the risk of PU occurrence coincided with the highest incidences of PUs recorded (14.6% in 2019 and 20.4% in 2020), indicating that as patient age and related risk factors increased, so did the incidence of PUs. This correlation emphasizes the critical need for tailored preventive strategies, particularly for older patients who may be more susceptible due to factors such as reduced mobility, comorbidities, and prolonged hospitalization.

Among the 143 patients who developed PUs in the ICU, a significant majority were male, comprising 67.8% of the cases (see Table 3). The average age of these patients was 64.9 years, which underscores the complexity of PU management in a population that is not only aging but also presents a multitude of health challenges. Notably, 95.8% of these patients were assessed as high risk for PU development according to the Braden scale, a standard tool for evaluating PU risk.

This high-risk profile highlights the vulnerability of this patient population, reinforcing the urgent need for comprehensive preventive strategies and diligent monitoring to reduce the occurrence and progression of pressure-related skin injuries within the ICU setting. By addressing the unique needs of this demographic, healthcare providers can implement more effective interventions that not only mitigate the risk of PUs but also enhance overall patient outcomes during critical care.

Table 4 shows the distribution of PUs across different anatomical sites in the ICU from 2017 to 2023. The sacral region was the most affected site, increasing from 16.7% in 2017 to 24.2% in 2020, likely due to prone positioning in COVID-19 patients. The heel area also saw significant variation, rising to 35.5% in 2021. In 2022, the head/forehead/face region accounted for 21.1% of PUs, likely due to prolonged unconsciousness or device use.

Other sites, like the malleolus and ear, showed fluctuating trends, with a surge in malleolus PUs to 50.0% in 2020 and ear PUs increasing to 36.4%, potentially related to medical equipment. Trochanter and buttock PUs occurred sporadically.

It is important to note that the anatomical site of PU development is not independent of the year of occurrence. The statistical analysis showed a significant relationship between the year and PU location (χ^2^(60) = 83.172; *p* = 0.026), indicating that evolving care practices and patient characteristics influenced PU distribution. 

The analysis of Table 5 reveals that systemic diseases, sensory deficits, and moisture emerged as consistent intrinsic risk factors, while pressure itself was identified as a significant extrinsic factor. These findings underscore the multifaceted nature of PU development, where both patient characteristics and environmental influences play crucial roles.

In particular, the intrinsic risk factors displayed a noteworthy correlation with age during the years 2019 and 2020. The incidence rates of PUs associated with age were 23.3% and 26.0%, respectively, indicating a substantial increase in vulnerability among older patients during these critical years. This relationship (χ^2^(6) = 16.8969; *p* = 0.010) suggests that age-related physiological changes (such as decreased skin elasticity and comorbid conditions) exacerbate the risk of developing PUs. The growing number of elderly patients in the ICU during this time could explain this trend, as this demographic often faces multiple health challenges that heighten their susceptibility to skin breakdown.

Regarding extrinsic risk factors, a significant relationship was observed with shear, particularly in 2019 and 2020, with shear rates consistently recorded at 22.2% (χ^2^(6) = 16.8969; *p* = 0.010). Shear forces can significantly contribute to tissue damage, especially in patients with limited mobility or those subjected to frequent repositioning. The heightened awareness of shear as a risk factor during these years may be attributed to evolving clinical practices and a greater emphasis on comprehensive PU prevention strategies.

Although the remaining risk factors did not reveal statistically significant relationships, their presence remains critical to understanding the full scope of PU development.

The analysis of Table 6 indicates that regarding the implemented measures, all the analyzed variables are not independent of the years in question. The implementation of risk and nutritional assessments varied significantly over the years, highlighting critical gaps in patient care during a particularly challenging period. Notably, the years 2019 and 2020 showed a higher proportion of risk assessments that were not conducted, reaching 26.7% (χ^2^(6) = 4.398; *p* = 0.623). This trend is concerning, as it suggests that a substantial number of patients in the ICU were not adequately evaluated for their risk of developing PUs. 

Additionally, the data reveals a significant increase in the proportion of unperformed nutritional assessments in 2020, with 24.7% not being conducted (χ^2^(6) =20.074; *p* = 0.003). Nutrition plays a vital role in skin health and healing, particularly for critically ill patients who are already at high risk for PUs. Inadequate nutritional assessment can impede the ability to identify patients who may benefit from specialized dietary interventions or supplementation, further increasing their susceptibility to skin breakdown. The lack of nutritional evaluations during a period when many patients faced heightened metabolic demands due to illness underscores the importance of integrating nutritional care into the overall treatment plan.

The analysis revealed that risk assessment and pressure control measures were consistently implemented throughout the studied periods, underscoring the commitment of the healthcare team to address the challenge of PUs in the ICU. However, despite these efforts, the results indicate that the incidence of PUs remains a significant clinical challenge, highlighting the need for continued vigilance and adaptation of strategies.

## 4. Discussion

The primary objective of this comprehensive study was to analyze the epidemiological changes and trends in the incidence and prevalence of PUs within the ICU context over the period from January 2017 to June 2023. Additionally, the study aimed to characterize the clinical profiles and risk factors of critically ill patients who developed PUs during their ICU hospitalization, as well as to evaluate the preventive measures that were implemented to mitigate the occurrence of these skin injuries. By providing these significant insights, the study offers a detailed understanding of the burden of PUs among ICU-hospitalized patients, particularly those who experienced PU development during their ICU stay.

The findings revealed an overall declining trend in the incidence of PUs in the ICU from 2017 to 2022, with a slight increase observed in 2023. This decline could be attributed to heightened awareness and the systematic implementation of evidence-based preventive strategies within the ICU setting, such as regular skin assessments and the use of advanced support surfaces. This trend mirrors global data [22], which reported an overall PU prevalence of 26.6%, with 16.2% within ICUs. Similar to our study, these data highlighted regional variations in PU prevalence, underlining the persistent and universal challenge PUs present in critical care settings. However, despite this overall decline, the persistence of PU incidence—even with a declining trend—signals the need for ongoing vigilance. This finding calls for the continued optimization of prevention protocols to further reduce the burden of these debilitating skin injuries in critically ill patients. The referenced study [22] corroborates this challenge, revealing that approximately 60% of ICU patients developed PUs regardless of regional prevalence. This underscores the importance of adopting preventive strategies that are not only comprehensive but also tailored to the specific context and conditions of each ICU setting, ensuring that prevention efforts are both effective and sustainable in reducing the incidence of PUs.

In terms of PU incidence and prevalence, the literature emphasizes the importance of precise and widely accessible data at local and national levels, offering a comprehensive understanding of PUs patterns in different contexts [22,23,24,25]. In our analyzed cohort, the PU prevalence rate was lower than that reported in other studies [1,8,22]. The incidence rate showed fluctuations over the years, with a notable peak in 2020, followed by a decreasing trend. The incidence rates observed in our study are lower than those reported in other international studies conducted in similar settings, where incidence rates ranged from 7.92% to 12.8%, and cumulative incidence rates from 10% to 25.9% [1,26,27].

These variations in PU prevalence and incidence rates may be attributed to several factors, including the level of knowledge and skills among nurses, the intensity of team surveillance and monitoring, and adherence to prevention protocols [1,23,28]. These factors play a crucial role in shaping outcomes, underscoring the need for continuous education and adherence to standardized preventive practices to further reduce the risk of PUs in ICU patients.

Although most patients in the cohort were male and aged over 65, these demographic factors alone may not be the strongest predictors of PU risk, as suggested by both national and international studies [29,30]. In fact, some research suggests that gender may not be a significant risk factor [29,30] for the development of PUs. Therefore, there is minimal evidence suggesting that gender is a risk factor associated with the development of PUs [29,30]. The literature emphasized that while sustained pressure over the skin is a primary cause of PUs, factors such as impaired mobility, sensation, nutrition, and natural skin aging play more significant roles. Our study supports this, indicating that patient characteristics like overall health status, comorbidities, and immobility are more influential in PU development than gender alone [9,23,26].

Regarding the relationship between gender, age, and PU occurrence risk, and despite certain trends identified in specific years, these are not consistent throughout the studied period, indicating annual variability that does not hold as a statistically significant pattern over the years. In 2019 and 2020, a higher incidence of PUs was observed among older individuals, highlighting their increasing vulnerability. This finding is consistent with a study who identified advanced age as a significant risk factor for medical device-related pressure injuries (MDRPIs) in ICU patients [31]. The physiological frailty and comorbidities commonly associated with aging, such as diabetes and peripheral vascular disease, exacerbate the risk of PU development. This is consistent with our observation of increased PU incidence among older patients in recent years, reinforcing the need for targeted preventive measures in this vulnerable population [1,22,31,32,33,34].

The results also emphasize the importance of considering not only individual characteristics, such as gender and age, but also temporal factors, such as the year, when analyzing PU development risk. This multifaceted approach can provide valuable information to identify specific periods where preventive measures can be intensified, helping to reduce the risk of PUs in vulnerable patients. Adapting prevention strategies to annual variations and different patient profiles is essential to improve the effectiveness of interventions and ensure more personalized and efficient care [6,23,35]. By examining both individual and temporal factors, healthcare providers can gain a more comprehensive understanding of the factors influencing PU development, allowing them to tailor prevention strategies to the unique needs of each patient and the evolving care environment. This holistic perspective can lead to more targeted and effective interventions, ultimately reducing the burden of PUs among critically ill patients in the intensive care setting.

More than 90% of the study sample was identified as high risk for PU development during the risk assessment conducted at the time of PU registration/identification in the ICU context. This high-risk classification contrasts with lower percentages reported in other settings, such as the medical and surgical departments of a Portuguese hospital, where 27.1% of patients were classified as high risk [24]. This discrepancy may stem from the ICU-specific challenges that elevate PU risk beyond general inpatient populations.

While PU risk assessment using tools such as the Braden Scale is a key component of clinical practice, as recommended by various guidelines and quality standards, our study reveals notable limitations [21,23,24,36,37]. Research suggests that the Braden Scale, being a generic tool, does not fully account for the unique physiological conditions of critically ill patients, potentially leading to an overestimation of PU risk [5,9,36,38,39]. Additionally, studies [31] have shown that factors such as elevated SOFA and APACHE II scores, extended use of medical devices, and prone positioning are crucial predictors of PUs that the Braden Scale may not adequately capture. Consequently, relying solely on this scale in ICU settings could hinder the optimal allocation of resources for PUs prevention [5,9,36,38,39].

To overcome these limitations, integrating ICU-specific risk assessment protocols alongside the Braden Scale could improve predictive accuracy and enable more targeted preventive measures. Modifying risk assessment tools to incorporate ICU-specific factors, as indicated by current research, may offer a more comprehensive evaluation of PU risk in this critical care environment [9,21,38,39,40].

Regarding the categorization and anatomical location of PUs acquired in the ICU, our study’s results are supported by previous studies conducted nationally [24,40] and internationally [1,22,26,41] in ICUs. The findings on the most frequently affected areas suggest that a significant proportion of ICU patients spend prolonged periods in dorsal, Fowler, or semi-Fowler positions, which increases pressure on specific points, particularly the sacral region and heels [1,9,40]. This sustained pressure on bony prominences, especially in immobile patients, significantly heightens the risk of developing PUs in these areas. The association between prolonged positioning and increased PU risk highlights the critical need for targeted interventions to relieve pressure on these vulnerable zones.

During the COVID-19 pandemic, the widespread use of prone positioning was expected to increase PUs in anterior regions such as the nose, face, and ears. However, our study found that PUs in these areas were less frequent than anticipated. This observation contrasts with some studies on COVID-19 patients [14,42,43,44,45,46,47], which reported higher incidences of anterior PUs due to prolonged prone positioning and the use of medical devices like oxygen masks and ventilation equipment. The discrepancy may be due to effective implementation of preventive measures such as enhanced padding and repositioning protocols, or it may reflect variations in study populations and ICU practices.

The categorization and anatomical location of PUs are essential for assessing severity and differentiating PUs from other skin lesions. However, this process remains subjective and influenced by the disparity of knowledge among nurses, even when following international guidelines [6,19,23]. These PU characteristics, along with incidence data, underscore important aspects of prevention, particularly the knowledge and skills of nurses, patient positioning, and workload. While recommendations on the frequency of repositioning critically ill patients remain debated, the data do not provide definitive conclusions on these factors [3,41,48,49]. Notably, the significant association between the year and the site of PU development suggests that changes in care practices—such as those brought about by the COVID-19 pandemic, procedural changes, or evolving patient demographics in ICUs—may have contributed to these variations [14,42,43]. This underscores the need to tailor preventive strategies to the specific circumstances of each period and focus on the most frequently affected areas. Adapting approaches to the evolving challenges in ICU care is crucial for effective PU prevention.

The analysis of the Portuguese ICU indicates that the structural changes, particularly the increase in ICU beds in 2020 during COVID-19 pandemic, likely had a significant impact on the staff’s ability to consistently implement comprehensive PUs prevention measures, as mentioned in another study [33]. With a higher patient-to-staff ratio, the increased workload and care complexity may have hindered the regular application of PU prevention measures. This highlights the need for not only protocols but also adequate staff-to-patient ratios to ensure proper care.

The introduction of specialized pressure-relieving mattresses in 2021 emerged as a pivotal intervention, contributing to a notable reduction in PUs occurrences. These mattresses, engineered to redistribute pressure and minimize skin damage, represent an essential component of PU prevention in critically ill patients [50,51,52]. Additionally, the implementation of evidence-based PU prevention bundles has shown a significant decrease in hospital-acquired pressure injuries (HAPIs) within critical care settings [53]. Specifically, one study reported a reduction in the HAPI index from 3.4 pre-intervention to 0.48 post-intervention over a period of 10 months, underscoring the effectiveness of bundled interventions that include staff training, standardized protocols, and supportive equipment [53].

Recent research has emphasized the importance of selecting, assessing, and managing support surfaces to effectively reduce the incidence of PUs and enhance the quality of care. These recommendations support the findings of our study regarding pressure-relieving mattresses, further confirming their positive role in preventing PUs.

The success of these initiatives underscores the importance of investing in ICU infrastructure and technology to enhance effective pressure ulcer prevention strategies.

Furthermore, the analysis identifies several key intrinsic and extrinsic risk factors for PU development. Systemic diseases, sensory deficits, and moisture were consistently identified as intrinsic risk factors, while pressure was noted as a significant extrinsic factor. Among these, age and shear forces emerged as critical predictors of PU occurrence in ICUs, as highlighted by studies conducted in other regions [1,34,54]. This reinforces the need for targeted interventions to address these risk factors, such as specialized care for elderly patients and measures to reduce shear forces on the skin [23,31,34]. A recent before-and-after study conducted in Brazil showed that pressure impacts skin temperature and moisture in the heels, critical factors in the development of PUs [55]. The results highlight the importance of pressure relief and moisture control in preventing these injuries, emphasizing the need for comprehensive preventive measures, as addressed in our study.

One of the identified gaps was the inconsistency in risk assessments during 2019 and 2020, as well as the lack of nutritional assessment in 2020. These evaluations are crucial for identifying at-risk patients and implementing timely interventions. It remains crucial to ensure that all preventive aspects are applied systematically and comprehensively to enhance the effectiveness of PU prevention strategies [22,23]. The absence of a specific PU prevention protocol likely contributed to these inconsistencies, underscoring the need for a systematic approach to preventive measures [26].

The results suggest the need for a continuous and detailed approach, highlighting the importance of access to precise and comprehensive data for a better understanding of the magnitude of the problem. These aspects are fundamental for identifying consistent patterns and improving PU prevention strategies [23]. The annual variability in PU development sites and the identified risk factors indicate the importance of adapting prevention strategies to the specific conditions of each period. Additionally, the failures in implementing preventive measures, especially in nutritional assessment, highlight critical areas that need improvement to reduce PU incidence.

Therefore, it is considered that a continuous and detailed approach, supported by precise and comprehensive data, is essential to efficiently address the PU problem. This will enable better adaptation of prevention strategies to the specific conditions of each period, the identification and correction of critical failures, and a clear distinction between preventable and unavoidable lesions, especially in the ICU context.

## 5. Limitations

The study’s observational nature allowed for the identification of associations between various factors and the development of Pus; however, it did not permit definitive conclusions regarding causal relationships. Additionally, since this research was conducted in the ICU of a single hospital, the findings may not be generalizable to other ICU settings, limiting broader applicability.

It is important to note that some PUs, particularly in an ICU context, are recognized as unavoidable due to the inherent complexities and vulnerabilities of critically ill patients [6,19,23,41]. However, the methodology of this study did not enable the researchers to distinguish between PUs that could have been prevented with appropriate measures and those that were unavoidable due to specific ICU risk factors. Understanding this distinction is essential for developing targeted prevention and care strategies that could enhance patient outcomes in ICU environments.

Another significant limitation was the inability to access and analyze data for the second half of 2023, due to a cyberattack on the healthcare institution. This gap in data analysis compromises the accuracy and representativeness of the study’s findings for that year, as the sample may not fully reflect the realities of patient care during this period. Additionally, the absence of data hinders a comprehensive understanding of temporal trends, making it difficult to draw extensive conclusions regarding the progression of PUs over time.

The lack of a standardized prevention protocol in the ICU may have also influenced the results. Without a consistent framework for assessing risk and implementing preventive measures, variations in practice could lead to discrepancies in PU incidence and prevalence. This absence could obscure the effectiveness of different interventions and complicate efforts to identify best practices in PU prevention.

In summary, while the study provides valuable insights, addressing these limitations through a more robust methodology—including a standardized prevention protocol and a comprehensive data collection strategy—would significantly enhance the reliability and applicability of the findings.

## 6. Implications for Practice and Future Research

One of the key strengths of this study lies in its direct clinical relevance. The results offer valuable insights that can be immediately applied in ICU settings to improve care and prevent PUs. By highlighting existing barriers to effective PU prevention, such as the need to enhance nurses’ knowledge and awareness, the study underscores the importance of overcoming these challenges to elevate the quality of care and ensure patient safety.

The findings not only inform clinical practice but also contribute significantly to the broader understanding of effective PU prevention strategies in critical care environments. This research provides a detailed analysis of six years of epidemiological trends (2017–2023), offering a comprehensive perspective on PU occurrence in ICUs over time. Such longitudinal data enables a more robust evaluation of changes and patterns in PU incidence and prevalence.

Additionally, the study critiques the limitations of the Braden Scale, commonly used for PU risk assessment, particularly in the context of critically ill patients. It suggests the need for more specific risk assessment tools tailored to ICU settings, where patient conditions are more complex.

The research also highlights the subjectivity involved in wound assessments due to variations in nurses’ knowledge and perception, despite the use of standardized international guidelines. This recognition points to the critical need for ongoing education and training in wound care to ensure consistent and accurate assessments.

The study’s findings, which indicate PU prevalence and incidence rates lower than those reported in other international studies, provide valuable comparative insights. These results highlight differences in PU rates across geographical and institutional contexts, reinforcing the importance of tailored prevention strategies. Moreover, the study identifies the positive impact of structural changes in the ICU—such as the implementation of specialized mattresses in 2021—on the reduction of PU occurrences. These improvements suggest that enhancing ICU infrastructure plays a key role in mitigating PU risks.

## 7. Conclusions

This six-year study provides essential insights into the epidemiology of PUs in a critical care setting. Our analysis revealed significant annual fluctuations in PU prevalence, ranging from 2.48% to 11%, underscoring the necessity for continuous surveillance and adaptable prevention strategies. The incidence rate varied between 2.54% and 5.71%, peaking in 2020, likely due to the COVID-19 pandemic and increased patient acuity, which highlights the influence of external factors on PU development. The sacrum and heels were the most affected areas, with Category 2 PUs being most frequent.

These findings underscore the importance of a multidisciplinary and proactive approach to PUs prevention in the ICU. Early identification of at-risk patients is crucial, particularly for those who are elderly, have systemic diseases, possess sensory deficits, or are susceptible to moisture. Regular and personalized assessments, alongside appropriate pressure relief techniques and nutritional monitoring, are vital components of effective prevention strategies.

Furthermore, this research highlights the urgent need for continuous education programs tailored for ICU nursing teams. By staying updated on best practices for PUs prevention, assessment, and treatment, healthcare providers can significantly reduce PU incidence and enhance patient outcomes.

To further strengthen the impact of this study, we recommend the implementation of dedicated PU prevention protocols to standardize care practices. Additionally, targeted educational programs for ICU nurses should be developed to enhance their knowledge and skills in PU management. Future research should prioritize the creation of risk assessment tools specifically designed for critically ill patients and further investigate the impact of external factors on PU development. By addressing these recommendations, we can improve the quality of care provided to patients in critical care settings.

## Figures and Tables

**Table 1:** Distribution of Gender, Age, PU Risk, Origin, and Number of ICU PUs per Patient over the confirmed!Analyzed Years.

	2017 *n* (%)	2018 *n* (%)	2019 *n* (%)	2020 *n* (%)	2021 *n* (%)	2022 *n* (%)	2023 *n* (%)
Sex							
Male	29 (67.4)	30 (63.8)	43 (82.7)	36 (66.7)	16 (69.6)	10 (45.5)	10 (62.5)
Female	14 (32.6)	17 (36.2)	9 (17.3)	18 (33.3)	7 (30.4)	12 (54.5)	6 (37.5)
Age	66.23 ± 13.61	62.06 ± 12.17	70.08 ± 12.20	64.00 ± 16.71	65.74 ± 15.30	67.64 ± 14.97	63.25 ± 21.19
Risk of developing PUs							
Not recorded	7 (16.3)	4 (8.5)	2 (3.8)	0 (0.0)	0 (0.0)	0 (0.0)	0 (0.0)
High risk	36 (83.7)	42 (89.4)	49 (94.2)	54 (100)	23 (100)	22 (100)	16 (100)
Low risk	0 (0.0)	1 (2.1)	1 (1.9)	0 (0.0)	0 (0.0)	0 (0.0)	0 (0.0)
Origin of PUs							
Not recorded	5 (9.6)	0 (0.0)	2 (2.9)	0 (0.0)	0 (0.0)	0 (0.0)	0 (0.0)
ICU	28 (53.8)	36 (52.2)	26 (37.1)	32(48.5)	26 (70.3)	20 (71.4)	13 (56.5)
Home	5 (9.6)	10 (14.5)	8 (11.4)	1 (1.5)	4 (10.8)	8 (28.6)	4 (17.4)
Other services	14 (26.9)	23 (33.3)	34 (48.6)	33 (50.0)	7 (18.9)	0 (0.0)	6 (26.1)
Number of Pus per patient in ICU							
1 PU	20 (83.3)	15 (57.7)	14 (66.7)	26 (92.9)	12 (66.7)	14 (82.4)	5 (55.6)
2 PUs	2 (8.3)	9 (34.6)	4 (19.0)	1 (3.6)	4 (22.2)	3 (17.6)	4 (44.4)
3 PUs	2 (8.3)	2 (7.7)	3 (14.3)	0 (0.0)	2 (11.1)	0 (0.0)	0 (0.0)
4 PUs	0 (0.0)	0 (0.0)	0 (0.0)	1 (3.6)	0 (0.0)	0 (0.0)	0 (0.0)

**Table 2:** Incidence and Prevalence PUs in the ICU.

	2017 (%)	2018 (%)	2019 (%)	2020 (%)	2021 (%)	2022 (%)	2023 (%)
Prevalence	7.86	8.39	8.84	11.0	3.57	3.48	4.52
Incidence	4.39	4.64	3.57	5.71	2.80	2.69	2.54

**Table 3:** Relationship between sex, age and risk of developing PU in ICU with the years under analysis.

	2017 *n* (%)	2018 *n* (%)	2019 *n* (%)	2020 *n* (%)	2021 *n* (%)	2022 *n* (%)	2023 *n* (%)	*p*-Value *
Sex								
Male	17 (17.5)	16 (16.5)	19 (19.6)	19 (19.6)	12 (12.4)	8 (8.2)	6 (6.2)	0.180
Female	7 (15.2)	10 (21.7)	2 (4.3)	9 (19.6)	6 (13.0)	9 (19.6)	3 (6.5)	
Age								
<60 years	7 (15.9)	11 (25.0)	6 (13.6)	6 (13.6)	4 (9.1)	5 (11.4)	5 (11.4)	0.575
[60; 71] years	9 (17.6)	10 (19.6)	6 (11.8)	11 (21.6)	8 (15.7)	4 (7.8)	3 (5.9)	
≥72 years	8 (16.7)	5 (10.4)	9 (18.8)	11 (22.9)	6 (12.5)	8 (16.7)	1 (2.1)	
Risk of developing PU								
Not registered	3 (50.0)	2 (33.3)	1 (16.7)	0 (0.0)	0 (0.0)	0 (0.0)	0 (0.0)	0.232
High risk	21 (15.3)	24 (17.5)	20 (14.6)	28 (20.4)	18 (13.1)	17 (12.4)	9 (6.6)	

Legend: * Chi-square test.

**Table 4:** Anatomical location of PUs over the years under analysis.

Variables	2017	2018	2019	2020	2021	2022	2023	*p*-Value *
Sacrum	11 (16.7)	11 (16.7)	9 (13.6)	16 (24.2)	7 (10.6)	7 (10.6)	5 (7.6)	0.026
Heel	2 (6.5)	7 (22.6)	4 (12.9)	1 (3.2)	11 (35.5)	2 (6.5)	4 (12.9)	
Trochanter	5 (45.5)	3 (27.3)	0 (0.0)	2 (18.2)	0 (0.0)	1 (9.1)	0 (0.0)	
Nose	3 (21.4)	4 (28.6)	1 (7.1)	3 (21.4)	1 (7.1)	2 (14.3)	0 (0.0)	
Malleolus	1 (25.0)	0 (0.0)	1 (25.5)	2 (50.0)	0 (0.0)	0 (0.0)	0 (0.0)	
Dorsal trunk region	2 (66.7)	1 (33.3)	0 (0.0)	0 (0.0)	0 (0.0)	0 (0.0)	0 (0.0)	
Head/forehead/face	2 (10.5)	4 (21.1)	4 (21.1)	1 (5.3)	2 (10.5)	4 (21.1)	2 (10.5)	
Ear	1 (9.1)	1 (9.1)	3 (27.3)	4 (36.4)	1 (9.1)	1 (9.1)	0 (0.0)	
Neck/scapular region	1 (20.0)	0 (0.0)	1 (20.0)	0 (0.0)	1 (20.0)	0 (0.0)	2 (40.0)	
Buttock	0 (0.0)	5 (33.3)	3 (20.0)	1 (6.7)	3 (20.0)	3 (20.0)	0 (0.0)	
Leg	0 (0.0)	0 (0.0)	0 (0.0)	2 (100.0)	0 (0.0)	0 (0.0)	0 (0.0)	

Legend: * Chi-square test.

**Table 5:** Distribution and relationship between extrinsic and intrinsic risk factors with the periods under analysis.

Variables			2017	2018	2019	2020	2021	2022	2023	*p*-Value *
Intrinsic risk factors	Age	Yes	8 (11.0)	10 (13.7)	17 (23.3)	19 (26.0)	8 (11.0)	8 (11.0)	3 (4.1)	0.010
		No	16 (22.9)	16 (22.9)	4 (5.7)	9 (12.9)	10 (14.3)	9 (12.9)	6 (8.6)	
	Weight	Yes	11 (16.9)	8 (12.3)	12 (18.5)	12 (18.5)	11 (16.9)	7 (10.8)	4 (6.2)	0.493
		No	13 (16.7)	18 (23.1)	9 (11.5)	16 (20.5)	7 (9.0)	10 (12.8)	5 (6.4)	
	Systemic diseases	Yes	11 (12.6)	11 (12.6)	14 (16.1)	22 (25.3)	12 (13.8)	12 (13.8)	5 (5.7)	0.087
		No	13 (23.2)	15 (26.8)	7 (12.5)	6 (10.7)	6 (10.7)	5 (8.9)	4 (7.1)	
	Malnutrition/Dehydration	Yes	9 (17.6)	3 (5.9)	12 (23.5)	13 (25.5)	5 (9.8)	6 (11.8)	3 (5.9)	0.047
		No	15 (16.3)	23 (25.0)	9 (9.8)	15 (16.3)	13(14.1)	11 (12.0)	6 (6.2)	
	Edema	Yes	12 (17.6)	15 (22.1)	10 (14.7)	12 (17.6)	7 (10.3)	8 (11.8)	4 (5.9)	0.924
		No	12 (16.0)	11 (14.7)	11 (14.7)	16 (21.3)	11 (14.7)	9 (12.0)	5 (6.7)	
	Sensory deficits	Yes	15 (16.3)	18 (19.6)	17 (18.5)	21 (22.8)	7 (7.6)	9 (9.8)	5 (5.4)	0.103
		No	9 (17.6)	8 (15.7)	4 (7.8)	7 (13.7)	11 (21.6)	8 (15.7)	4 (7.8)	
	Moisture	Yes	16 (18.6)	17 (19.8)	13 (15.1)	18 (20.9)	10 (11.6)	5 (5.8)	7 (8.1)	0.175
		No	8 (14.0)	9 (15.8)	8 (14.0)	10 (17.5)	8 (14.0)	12 (21.1)	2 (3.5)	
	Temperature	Yes	10 (18.9)	11 (20.8)	6 (11.3)	16 (30.2)	4 (7.5)	4 (7.5)	2 (3.8)	0.129
		No	14 (15.6)	15 (16.7)	15 (16.7)	12 (13.3)	14 (15.6)	13 (14.4)	7 (7.8)	
Extrinsic risk factors	Pressure	Yes	23 (17.8)	24 (18.6)	16 (12.4)	27 (20.9)	15 (11.6)	15 (11.6)	9 (7.0)	0.179
		No	1 (7.1)	2 (14.3)	5 (35.7)	1 (7.1)	3 (21.4)	2 (14.3)	0 (0.0)	
	Friction	Yes	17 (20.5)	18 (21.7)	15 (18.1)	15 (18.1)	9 (10.8)	5 (6.0)	4 (4.8)	0.071
		No	7 (11.7)	8 (13.3)	6 (10.0)	13 (21.7)	9 (15.0)	12 (20.0)	5 (8.3)	
	Shear	Yes	6 (13.3)	4 (8.9)	10 (22.2)	10 (22.2)	6 (13.3)	3 (6.7)	6 (13.3)	0.042
		No	18 (18.4)	22 (22.4)	11 (11.2)	18 (18.4)	12 (12.2)	14 (14.3)	3 (3.1)	

Legend: * Chi-square test.

**Table 6:** Distribution and relationship of implemented measures by periods under analysis.

Variables		2017	2018	2019	2020	2021	2022	2023	*p*-Value *
Risk assessment	Yes	23 (18.0)	24 (18.8)	17 (13.3)	24 (18.8)	16 (12.5)	15 (11.7)	9 (7.0)	0.623
	No	1 (6.7)	2 (13.3)	4 (26.7)	4 (26.7)	2 (13.3)	2 (13.3)	0 (0.0)	
Moisture control	Yes	18 (20.0)	23 (25.6)	15 (16.7)	14 (15.6)	9 (10.0)	8 (8.9)	3 (3.3)	0.006
	No	6 (11.3)	3 (5.7)	6 (11.3)	14 (26.4)	9 (17.0)	9 (17.0)	6 (11.3)	
Pressure control	Yes	24 (19.8)	24 (19.8)	18 (14.9)	24 (19.8)	13 (10.7)	12 (9.9)	6 (5.0)	0.051
	No	0 (0.0)	2 (9.1)	3 (13.6)	4 (18.2)	5 (22.7)	5 (22.7)	3 (13.6)	
Nutritional assessment	Yes	15 (32.6)	11 (23.9)	8 (17.4)	4 (8.7)	4 (8.7)	3 (6.5)	1 (2.2)	0.003
	No	9 (9.3)	15 (15.5)	13 (13.4)	24 (24.7)	14 (14.4)	14 (14.4)	8 (8.2)	

Legend: * Chi-square test.

## Data Availability

Dataset available on request from the authors.

## Public Involvement Statement

No public involvement in any aspect of this research.

## Guidelines and Standards Statement

This manuscript was drafted against the Strengthening the Reporting of Observational studies in Epidemiology (STROBE) Statement for cohort studies.
